# Barriers to pressure injury prevention and associated factors among critical care nurses in Malaysia

**DOI:** 10.1186/s12912-025-03963-4

**Published:** 2025-10-21

**Authors:** Muamar Iskandar Mohamed Yusoff, Mohd Ismail Ibrahim

**Affiliations:** Department of Community Medicine, School of Medical Sciences, Health Campus, Universiti Sains Malaysia, Kubang Kerian, Kota Bharu, Kelantan 16150 Malaysia

**Keywords:** Pressure injury, Critical care, Nurses, Patient safety, Barriers, Guideline access

## Abstract

**Background:**

Pressure injury (PI) remains a major patient safety concern in intensive care, where immobility, high acuity, and workload increase risk. Although guidelines exist, prevention practices in Malaysian tertiary hospitals are inconsistent. Understanding perceived barriers and associated factors among critical care nurses is essential for targeted interventions.

**Methods:**

A cross-sectional study was conducted among registered nurses in ICUs, NICUs, PICUs, HDUs, and CCUs across three tertiary hospitals in Perak, Malaysia. Stratified random sampling proportionate to hospital and unit size selected 384 nurses, of which 347 completed the survey (response rate 90.4%). Data were collected using a validated 25-item Pressure Injury Prevention Barriers (PIPB) questionnaire (Cronbach’s α = 0.90). Descriptive statistics summarized characteristics and barrier scores, while logistic regression identified factors associated with high perceived barriers (*p* < 0.05).

**Results:**

Barriers to PI prevention were highly prevalent. The main barriers were frequent staff turnover (score = 99), poor cooperation from family caregivers (84), and lack of preventive devices (71). Multivariate analysis indicated that staff turnover, limited caregiver cooperation, and restricted access to guidelines were the strongest predictors of perceived barriers.

**Conclusions:**

PI prevention in Malaysian critical care units is hampered by staff turnover, limited caregiver cooperation, equipment shortages, and uneven access to guidelines. Strengthening guideline dissemination, retention policies, resource provision, and multidisciplinary engagement is vital to improve patient safety.

**Supplementary Information:**

The online version contains supplementary material available at 10.1186/s12912-025-03963-4.

## Introduction

A pressure injury (PI) is a localized area of skin and underlying tissue damage, typically occurring over bony prominences as a result of sustained pressure, shear, or friction [1–4]. They represent a major patient safety issue, leading to increased morbidity, prolonged hospitalization, higher healthcare costs, and reduced quality of life [5, 6]. Global estimates suggest hospital-acquired PI affect 12–18% of hospitalized adults, with higher rates in resource-constrained regions where staffing, infrastructure, and equipment availability are limited [7–9]. Device-related injuries, such as those from nasal cannulae or endotracheal tubes, are an additional concern in intensive care settings [10, 11].

In low- and middle-income countries (LMICs), including those in Southeast Asia, PI prevention is hampered by resource shortages, inconsistent staffing, and training gaps [12–15]. In Malaysia, national guidelines exist, yet implementation in critical care units remains inconsistent. The Malaysian Registry of Intensive Care (MRIC) reported 3.4–6.8 PI per 1000 ICU days in 2017, with wide institutional variation and issues of underreporting [16]. Local studies further highlight persistent barriers such as high staff turnover, limited access to preventive devices, and challenges in integrating guidelines into daily practice [15, 17–19].

Critical care units pose particular difficulties for prevention due to high patient acuity, sedation, mechanical ventilation, and time-intensive workflows [20–22]. Evidence shows that staffing ratios, workload, access to pressure-relieving devices, and ongoing training strongly influence preventive outcomes [23–25]. Even in well-resourced settings, sustainable prevention is difficult without organizational support and adequate resources [26, 27]. Moreover, recent research highlights that nurse well-being, organizational culture, and the physical work environment play crucial roles in compliance with safety protocols and patient outcomes, further compounding PI prevention challenges in LMIC settings [28, 29].

To address this gap, we applied Donabedian’s Quality of Care Model, which conceptualizes healthcare quality in terms of structure, process, and outcomes [30]. Structural factors include staffing levels, equipment availability, and access to guidelines; process factors cover training, teamwork, and engagement with family caregivers. In our study, perceived barriers represent the outcome, reflecting how these structural and process factors influence nurses’ capacity to implement prevention. This framework enables a context-specific understanding of PI prevention challenges in Malaysian tertiary hospitals and informs strategies relevant to other LMIC settings facing similar constraints [15–18, 30].

Therefore, this study aimed to identify perceived barriers to pressure injury prevention and to determine the associated sociodemographic and work-related factors among critical care nurses in tertiary hospitals in Perak, Malaysia.

## Methodology

### Study design and setting

This cross-sectional study was conducted from February to March 2024 across three tertiary hospitals in Perak, Malaysia. The hospitals included diverse critical care units such as intensive care units (ICUs), neonatal intensive care units (NICUs), pediatric intensive care units (PICUs), high dependency units (HDUs), and coronary care units (CCUs). This range ensured coverage of typical critical care environments within the Malaysian public healthcare system, supporting the study’s aim to capture barriers faced across these settings [31, 32].

### Study criteria

The study targeted registered nurses directly involved in patient care within critical care units of the participating hospitals. Nurses were required to have at least six months of continuous experience in their current critical care unit, ensuring sufficient familiarity with unit workflows, clinical routines, and pressure injury prevention practices. This criterion was set to avoid capturing responses from staff too new to have integrated into standard prevention protocols or unit culture. Nurses in purely managerial or administrative roles without bedside responsibilities, and those on extended leave during the data collection period, were excluded to ensure that only those actively engaged in daily patient care were surveyed.

### Participants and sampling

A stratified random sampling strategy was employed to ensure representative participation across hospitals and unit types. Each critical care unit within the hospitals served as a stratum, with proportional allocation reflecting the relative size of each unit. Eligible nurses were then randomly selected from official staff rosters supplied by hospital administrators. To protect confidentiality, rosters were anonymized by removing names and personal identifiers prior to selection. This process minimized selection bias, ensured fairness in recruitment, and safeguarded participant privacy [33].

### Sample size calculation

The minimum sample size was estimated using a two-proportion formula to detect a 15% difference in perceived barrier prevalence between nurses with and without access to guidelines. The calculation assumed a significance level of α = 0.05 and 80% power, resulting in a requirement of 348 participants. To accommodate a possible 10% non-response, the target sample was set at 384. Of the 384 nurses approached, 37 chose not to participate, resulting in a response rate of 90.4%. While this high response rate minimizes the risk of non-response bias, we acknowledge that selection bias cannot be fully excluded.

### Data collection instrument

Data were collected using the validated Pressure Injury Prevention Barriers (PIPB) questionnaire developed by Lopez-Franco et al. [34]. The PIPB comprises 25 items covering perceived barriers related to both structural and process dimensions, including workload, staffing levels, equipment availability, guideline access, training adequacy, and family cooperation. Items were rated on a 4-point Likert scale (1 = strongly disagree to 4 = strongly agree), with higher scores indicating stronger perceived barriers. Permission to use and adapt the questionnaire for local context was obtained from the original authors.

Minor contextual adaptations (such as updating local terminology) were implemented to improve relevance and clarity. For example, the term ‘skin care products’ was contextualized as ‘barrier creams commonly used in Malaysian hospitals,’ and complex phrasing was simplified to match local usage. A pretest was conducted with 20 critical care nurses in a non-participating hospital to evaluate clarity, cultural appropriateness, and relevance of the instrument. Feedback confirmed that the items were understandable and contextually suitable. Minor terminology adjustments were made (e.g., specifying ‘barrier creams commonly used in Malaysian hospitals’ instead of general ‘skin care products’). These changes supported the tool’s face and content validity in the local context, in addition to the high internal consistency observed in this study (Cronbach’s α = 0.90).

### Data collection procedure

Prior to data collection, institutional approvals were obtained from each participating hospital. Trained data collectors visited units during shift changes to distribute paper-based questionnaires, along with study information sheets outlining objectives, participant rights, and confidentiality assurances. Nurses willing to participate provided written informed consent and returned completed questionnaires anonymously into locked collection boxes placed in each unit. This method safeguarded confidentiality, minimized potential coercion, and accommodated shift work demands over a six-week collection period.

### Ethical consideration

Ethical approval for this study was obtained from the relevant committees, including the Jawatankuasa Etika Penyelidikan Manusia Universiti Sains Malaysia (JEPeM-USM) (Code: USM/JEPeM/KK/24111031) and the National Medical Research Register (NMRR) (ID: NMRR-24-03884-UTY [IIR]). Strict procedures were implemented to maintain participant confidentiality, and all data analysis and reporting were conducted without disclosing participant identities. Completed questionnaires were stored securely in locked cabinets accessible only to the research team, and electronic data were entered into a password-protected database with restricted access. All responses were anonymized prior to analysis to ensure confidentiality and protect participant identity.

### Data analysis

Data were analyzed using IBM SPSS version 29.0. Descriptive statistics, including means, standard deviations, frequencies, and percentages, were used to summarize participant characteristics and overall barrier scores. A total barrier score was calculated by summing item responses (range 25–100), with higher scores indicating greater perceived barriers.

Bivariate analyses were conducted using simple logistic regression to examine associations between high perceived barrier levels (dichotomized using the median split) and independent variables, such as age, gender, education level, years of experience, unit type, and access to guidelines. A p-value threshold of 0.25 was used for variable selection into the multivariate model. This threshold was deliberately chosen to reduce the risk of excluding potential confounders or predictors that might not appear significant in univariate analysis but could have meaningful effects when adjusted for other variables in multivariate analysis, consistent with recommended best practices for logistic regression modeling [35]. The use of the sample median as a cut-off ensured balanced groups for regression analysis and was consistent with prior studies employing the PIPB tool [27]. Although quartile-based or clinically validated thresholds may provide more nuanced categorization, such standards are not currently established, and sensitivity analyses may be warranted in future research.

Variables meeting this threshold were included in the multivariate logistic regression analysis to identify independent predictors of high perceived barriers. Results were presented as adjusted odds ratios (AORs) with 95% confidence intervals (CIs) and associated p-values. Model fit was evaluated using the Hosmer–Lemeshow goodness-of-fit test and the area under the receiver operating characteristic (ROC) curve to assess discriminative ability. We also acknowledge that unmeasured confounders such as workload intensity and unit leadership were not assessed in this study but may influence perceived barriers.

## Results

### Participant characteristics

From a total population of 598 registered critical care nurses across three tertiary hospitals in Perak, a stratified random sample of 384 nurses was drawn, proportionate to hospital and unit size. Of these, 347 completed the survey, yielding a response rate of 90.4%. Table 1 presents the sociodemographic profile of participants. The majority were female and below 40 years of age. Most held diplomas in nursing, while a smaller proportion possessed bachelor’s degrees. In terms of professional experience, most participants had fewer than 10 years of practice in critical care nursing.

**Table 1:** Sociodemographic characteristics of the participants (*n* = 347)

Characteristic	*n* (%)	Mean (SD)
Age*		
≤40 years old	226(65.1)	37.95(6.5)
>40 years old	121(34.9)	
Sex		
Male	15(4.3)	
Female	332(95.7)	
Race		
Malay	310(89.3)	
Chinese	7(2.0)	
Indian	21(6.1)	
Others	9(2.6)	
Level of education		
Diploma	292(84.2)	
Advanced Diploma	39(11.2)	
Degree	16(4.6)	
Master/PHD	0	
Working Duration		
≤5 years	64(18.4)	13.8(6.85)
>5 years	283(81.6)	
Hospital		
HRPB	187(53.9)	
HTPG	122(35.2)	
HSM	38(10.9)	
Current Working Unit		
ICU	106(30.5)	
PICU	46(13.2)	
NICU	130(37.5)	
HDU	37(10.7)	
CCU	28(8.1)	

*Sari et al., (2023)

### Working characteristics

Participants represented a range of professional designations, with staff nurses forming the largest group, alongside smaller numbers of senior staff nurses and charge nurses. Distribution across unit types was diverse, with the largest groups working in ICUs and NICUs. Access to PI prevention guidelines or literature varied, with just over half of the nurses reporting such access. Table 2 shows the summary of the working characteristics of the participants.

**Table 2:** Working characteristics of the participants (*n* = 347)

Characteristic	*n* (%)
Current Working Unit	
ICU	106(30.5)
PICU	46(13.2)
NICU	130(37.5)
HDU	37(10.7)
Nurse-to-patient ratio in your unit	
1:1	11(3.2)
1:2	210(60.5)
1:3	52(15.0)
≥1:4	74(21.3)
Do you have access to guidelines on managing Pressure Injury?	
Yes	280(80.7)
No	67(19.3)
Do you aware of NPUAP/EPUAP(National & European Pressure Ulcer Advisory Panel) International PI (pressure injury) Prevention and Treatment Guidelines?	
Yes	189(54.4)
No	158(45.6)
How many hours work averagely for one week?	
≤ 50 h	265(76.4)
>50 h	82(23.6)
How often do you manage PI (pressure injury) patient?	
Never	64(18.4)
Almost Never	87(25.1)
Sometimes	44(12.7)
Almost everyday	12(3.5)
Everyday	140(40.3)

### Perceived barriers to pressure injury prevention

All 25 barriers identified using the PIPB questionnaire are presented in Supplementary Table S1. In the main text, we highlight the top barriers most frequently reported by respondents. The barrier scores were calculated based on responses where participants selected ‘frequent’ or ‘always,’ indicating consistent challenges in practice. While the sample mean score was used as the cut-off to categorize participants into high and low perceived barrier groups for subsequent analyses, consistent with the validated scoring approach [27].

The three most highly rated barriers were:

- High in-service turnover of nursing staff (total score = 99).
- Lack of cooperation from family caregivers (score = 84).
- Unavailability of preventive devices (score = 71).

These findings highlight both systemic and interpersonal challenges faced by critical care nurses in implementing consistent PI prevention practices.

### Bivariate analysis of factors associated with perceived barriers

Bivariate analysis using simple logistic regression assessed associations between high perceived barrier levels (categorized by median split) and participant characteristics as summarized in Table 3. Results indicated access to PI prevention guidelines or literature (*p* = 0.007) and current working unit (*p* ≤ 0.25) met the inclusion threshold for multivariate analysis as shown in Table 4.

There were no significant associations between perceived barriers and sociodemographic variables such as age, gender, ethnicity, education level, or years of experience.

**Table 3:** Simple logistic regression analysis of associated factors of high barriers to pressure injury prevention among critical care nurses in tertiary hospitals, Perak

Variables	Crude OR (95% CI)	*p*-value
Age (years)	0.90 (0.58, 1.40)	0.643
Gender		
Male	Ref	
Female	0.61 (0.20, 1.81)	0.371
Race		
Non Malay	Ref	
Malay	0.94 (0.48, 1.87)	0.869
Education level		
Degree/ Masters	Ref	
Advance Diploma/Certificate		
Diploma		
Current Working Unit		
PICU	Ref	
NICU	1.82 (0.67, 4.93)	0.240
ICU	0.36 (1.00, 5.52)	0.049
HDU	1.28 (0.56, 2.96)	0.556
Frequency of managing PI		
Never	Ref	
Almost Never	0.82 (0.24, 2.78)	0.743
Sometimes	1.52 (0.46, 5.04)	0.488
Almost everyday	0.84 (0.24, 2.94)	0.785
Everyday	1.28 (0.35, 4.65)	0.709
Working experience (years)	0.97 (0.58,1.67)	0.909
Access to guideline/literature		
No	Ref	
Yes	2.19 (1.23, 3.88)	0.007
Working hours (total hours/ week)	0.96 (0.58, 1.58)	0.873
Nurse-to-patient ratio		
1:1	Ref	
1:2	0.85 (0.25, 2.87)	0.792
1:3 ≥ 1:4	0.88 (0.24, 3.26) 1.49 (0.42, 5.32)	0.848 0.538

### Multivariate logistic regression analysis

Variables meeting the *p* ≤ 0.25 criterion in bivariate analysis were entered into the multivariate logistic regression model to identify independent predictors of high perceived barriers. The final model revealed that nurses with access to PI prevention guidelines or literature had significantly lower odds of perceiving high barriers. Specifically, nurses who had access to clinical guidelines were 59% less likely to report high barriers to pressure injury prevention compared to those without access (AOR = 0.41, 95% CI: 0.22–0.79, *p* = 0.007).

Compared to nurses working in PICUs (reference group):

- NICU nurses were 82% less likely to perceive high barriers (AOR = 0.18, 95% CI: 0.08–0.43, *p* < 0.001).
- HDU nurses were 67% less likely to report high barriers (AOR = 0.33, 95% CI: 0.12–0.91, *p* = 0.032).
- ICU nurses did not show a statistically significant difference compared to PICU nurses (*p* = 0.064).

These results indicate that unit type and access to guidelines were important independent predictors of perceived barriers to PI prevention among critical care nurses.

**Table 4:** Multiple logistic regression analysis of associated factors of high barriers to pressure injury prevention among critical care nurses in tertiary hospitals, Perak

Variables	Crude OR (95% CI) ^a^	*p*-value	Adjusted OR (95% CI)^b^	*p*-value
Access to guideline/literature				
No	Ref			
Yes	2.19(1.23,3.88)	0.007	**0.41 (0.22**,**0.79)**	**0.007**
Current Working Unit				
PICU	Ref			
NICU	1.82 (0.67, 4.93)	0.240	**0.18 (0.08**,**0.43)**	**< 0.001**
ICU	0.36 (1.00, 5.52)	0.049	**0.43 (0.18**,**1.05)**	**0.064**
HDU	1.28 (0.56, 2.96)	0.556	**0.33 (0.12**,**0.91)**	**0.032**

^a^ Simple Logistic Regression

^b^ Multiple Logistic Regression

Constant 2.121

Forward LR method applied

No multicollinearity and no interaction

Hosmer and Lemeshow test, p value 0.942

undefinedClassification Table 62.2% correctly classified

Area under Receiver Operating Characteristics (ROC) 65%

### Model fit and predictive ability

The final multivariate logistic regression model demonstrated good fit, as indicated by a non-significant Hosmer–Lemeshow test (*p* = 0.942), suggesting the model adequately described the data. The model correctly classified 62.2% of cases. The area under the ROC curve (AUC) was 0.65, reflecting modest but acceptable discriminative ability in distinguishing between high and low perceived barrier levels.

## Discussion

This study examined perceived barriers to pressure injury (PI) prevention and associated factors among critical care nurses in tertiary hospitals in Perak, Malaysia. Perceived barriers were highly prevalent, with staffing issues, family-related challenges, and equipment shortages emerging as central obstacles. High in-service turnover was the most frequently cited barrier, reflecting systemic human resource challenges common in many low- and middle-income countries (LMICs). Reliance on contract staff and uneven workforce distribution exacerbate turnover, leading to instability, reduced continuity of care, and increased burnout [16, 17, 28, 36–38].

Lack of cooperation from family caregivers highlighted the interpersonal complexities of PI prevention in Malaysia, where family involvement in patient care is deeply embedded but often informal and unstructured [15, 18]. Resistance to repositioning or limited awareness of prevention measures complicates nursing workflows, echoing regional findings that structured caregiver education and engagement are necessary to transform families into effective partners [27, 28].

Resource shortages, particularly the limited availability of pressure-relieving mattresses, turning devices, and positioning aids, were also identified as major barriers. Similar procurement and budgetary challenges have been reported across LMICs [9, 12, 28]. Even with guidelines in place, prevention cannot succeed without adequate institutional commitment to supply essential equipment.

Guideline access emerged as a strong predictor of lower perceived barriers to pressure injury prevention. Nurses who had access to updated and practical guidelines reported fewer challenges, emphasizing the critical role of knowledge translation in clinical practice. Nevertheless, evidence indicates that guidelines alone are insufficient; their impact depends on effective dissemination, regular staff training, and seamless integration into daily workflows [8, 10, 13, 15, 24]. Our findings align with the broader literature on evidence-based practice. For instance, Jarrar et al. (2025) [39] demonstrated that structured guideline use combined with shared decision-making improved both clinical and psychosocial outcomes among patients with type 2 diabetes, highlighting the broader benefits of guideline-driven care. This parallel reinforces the need not only to develop evidence-based guidelines but also to ensure their accessibility, usability, and incorporation into routine nursing practice. Translating guidelines into everyday care is therefore a critical step in overcoming barriers and strengthening patient safety. Furthermore, organizational factors such as management support and psychological empowerment have been shown to enhance compliance with safety protocols [40], while structured guideline use and collaborative decision-making foster the consistent application of evidence-based care [41].

Unit type was another significant predictor. Nurses in NICU and HDU perceived fewer barriers compared to those in PICUs, likely due to clearer care protocols, smaller patient loads, and more standardized pathways [20, 23, 29, 36]. By contrast, PICUs manage complex, multisystem cases and rely heavily on family caregivers, which can complicate prevention [41]. These findings align with studies showing that organizational and demographic factors shape safety outcomes [42], and that person-centered, supportive practice environments improve quality and safety [36].

International evidence reinforces that barriers identified in Malaysia mirror those reported globally. Studies from Southeast Asia, Africa, and Latin America describe similar issues of staff shortages, resource constraints, and family-related challenges [27, 28, 43, 44]. The WHO and international nursing bodies emphasize coordinated strategies combining workforce capacity, equipment provision, training, and institutional policy [1, 3, 24].

Policy implications are clear. PI prevention should be incorporated into national patient safety indicators, supported by mandatory training modules, dedicated budgets for prevention devices, and structured caregiver education. Within Malaysia’s public health system, interventions such as in-service training, mentorship, rotational staffing, and ring-fenced budgets have already proven feasible in other quality improvement initiatives [29].

Finally, these findings are well interpreted through Donabedian’s Quality of Care Model. Structural factors (staffing levels, equipment availability, guideline access) define the system’s capacity for safe care. Process factors (training, teamwork, caregiver engagement) shape how preventive practices are delivered. The outcome which is nurses’ perceived barriers reflects the interplay of these domains. Applying Donabedian’s model highlights leverage points for intervention: strengthening structural supports, optimizing care processes, and thereby reducing barriers to PI prevention. This structured lens ensures that policy and practice recommendations are evidence-based and context-specific.

### Strength and limitation

This study has several notable strengths. The use of a validated instrument with strong internal consistency [33], a relatively large sample size, and stratified random sampling across diverse critical care units enhance the representativeness of the findings within the public tertiary hospital context. The application of a structured framework further strengthens the methodological rigor and ensures that both individual and organizational factors were considered.

Nevertheless, several limitations must be acknowledged. First, the cross-sectional design restricts the ability to draw causal inferences, thereby limiting the extent to which associations identified here can be directly translated into policy or practice. Second, reliance on self-reported measures may have introduced response biases, including recall errors and the tendency to provide socially desirable answers. Third, some potential for selection bias cannot be ruled out, particularly if unit managers had any influence on staff participation. Fourth, because the study was conducted only in public tertiary hospitals in Perak, the findings may not be generalizable to other settings such as private hospitals, district hospitals, or non-Malaysian contexts where organizational structures and resources differ. Prior studies have shown that hospital characteristics which include size, accreditation status, teaching designation, and nurse working conditions such as shift length that can significantly influence patient safety outcomes [38, 45], which underscores the need for caution when extrapolating these results to different healthcare systems. Finally, while categorizing barrier levels using the mean score is consistent with prior research [27], this approach may oversimplify nuanced variations in nurses’ perceptions of barriers.

## Conclusion

This study identified staff turnover, limited caregiver cooperation, and shortages of essential equipment as key systemic barriers to pressure injury prevention in Malaysian critical care settings. Access to clinical guidelines was shown to significantly reduce perceived barriers, emphasizing the importance of translating evidence into practice through effective knowledge dissemination and training. The findings suggest that prevention strategies should be tailored to the specific needs of different critical care units rather than adopting uniform, one-size-fits-all approaches. These recommendations are consistent with international best practices, including the WHO Global Patient Safety Action Plan 2021–2030 and the EPUAP/NPIAP/PPPIA International Guideline for the Prevention and Treatment of Pressure Ulcers/Injuries [1, 46]. Moving forward, integrating pressure injury prevention into national patient safety indicators, ensuring adequate staffing ratios, promoting nurse well-being, and empowering staff with accessible guidelines and resources are critical steps to overcoming barriers and improving patient outcomes.

## Supplementary Information

Below is the link to the electronic supplementary material.

Supplementary Material 1

## Data Availability

The datasets generated and/or analysed during the current study are not publicly available due to the need to maintain the anonymity of nurses and the confidentiality of the data. However, the datasets are available from the corresponding author on reasonable request.

## Abbreviations

PI: Pressure Injury
ICU: Intensive Care Unit
NICU: Neonatal Intensive Care Unit
PICU: Pediatric Intensive Care Unit
HDU: High Dependency Unit
CCU: Coronary Care Unit
CI: Confidence Interval
