# Supplementary material for: Barriers to pressure injury prevention and associated factors among critical care nurses in Malaysia
Source: BMC Nurs. 2025 Oct 21;24:1299. doi: 10.1186/s12912-025-03963-4 (PMC12542456; doi:10.1186/s12912-025-03963-4)
Supplement: Supplementary file 1 — Supplementary Material 1 [file 12912_2025_3963_MOESM1_ESM.docx]

Supplementary Table S1

Table SI: Pressure injury prevention barriers among critical care nurses at tertiary hospitals in Perak

| Barriers | Never | Sometimes | Frequently | Always | Total Score* |
| --- | --- | --- | --- | --- | --- |
| 1. Difficulty in understanding the recommendations and/or their level of evidence from guidelines or protocols. | 113 | 218 | 14 | 2 | 16 |
| 2. Lack of motivation among nursing professionals. | 158 | 171 | 14 | 4 | 18 |
| 3. Difficulty in understanding research findings. | 98 | 228 | 17 | 4 | 21 |
| 4. Low priority given to pressure injury prevention by nursing professionals. | 136 | 196 | 11 | 4 | 15 |
| 5. Lack of preventive devices (e.g., special mattresses, cushions, skin care products). | 79 | 197 | 49 | 22 | 71 |
| 6. Lack of knowledge regarding pressure injury prevention. | 175 | 161 | 6 | 5 | 11 |
| 7. Lack of time to provide preventive care. | 103 | 197 | 29 | 18 | 47 |
| 8. Incorrect use of equipment and devices due to inadequate staff training. | 125 | 195 | 21 | 6 | 27 |
| 9. Lack of evaluation by facility management of preventive interventions provided by the nursing team. | 112 | 210 | 19 | 6 | 25 |
| 10. Lack of job satisfaction among nursing professionals. | 109 | 205 | 26 | 7 | 33 |
| 11. Lack of patient cooperation in applying preventive measures. | 75 | 211 | 49 | 12 | 61 |
| 12. Lack of multidisciplinary team for prevention. | 86 | 205 | 41 | 15 | 56 |
| 13. Incomplete recording of the interventions provided to the patients. | 106 | 219 | 19 | 3 | 22 |
| 14. Difficulty in understanding pressure injury risk assessment scales. | 128 | 203 | 11 | 5 | 16 |
| 15. Lack of awareness of potential legal responsibilities when patients develop pressure injuries during hospital stay. | 183 | 146 | 16 | 2 | 18 |
| 16. Variability in implementation and poor adherence of care plans. | 110 | 212 | 19 | 6 | 25 |
| 17. Lack of communication within the multidisciplinary team. | 126 | 197 | 18 | 6 | 24 |
| 18. Difficulty to record the interventions in patients at risk of pressure injuries, due to the format of the form or register. | 130 | 169 | 43 | 5 | 48 |
| 19. Lack of protocols or clinical guidelines for pressure injuries prevention, in the workplace. | 140 | 172 | 32 | 3 | 35 |
| 20. Lack of training courses on pressure injury prevention available in the workplace. | 96 | 212 | 30 | 9 | 39 |
| 21. Lack of continuity of care across different settings (hospitals, nursing homes, primary care centres) | 70 | 219 | 47 | 11 | 58 |
| 22. Lack of cooperation from family caregivers at the hospital. | 62 | 201 | 58 | 26 | 84 |
| 23. Persistence of traditional, non-evidence-based, cares ("it has always been done this way") | 101 | 209 | 28 | 9 | 37 |
| 24. Lack of a clear definition of professional roles and responsibilities for pressure injury prevention. | 115 | 201 | 25 | 6 | 31 |
| 25. High turnover of nursing staff. | 70 | 178 | 64 | 35 | 99 |

***Total score= Frequently + Always**

Supplementary Table S1 presents the full item-level distribution of responses to the Pressure Injury Prevention Barriers Scale among critical care nurses in tertiary hospitals in Perak. The table details the frequency of each response option (Never, Sometimes, Frequently, Always) across all 25 barrier items, along with total scores, calculated by combining the “Frequently” and “Always” categories. This supplementary material allows readers to examine the detailed response patterns underlying the aggregated findings in the main text, providing transparency on how participants rated individual barriers, such as lack of equipment, family cooperation, and staff turnover.
